# Effects on Soil Fertility and Crop Productivity Under Residual Agricultural Gypsum and *Azospirillum brasilense* in Cover Crops in a Consolidated No-Tillage System

**DOI:** 10.3390/plants14203230

**Published:** 2025-10-21

**Authors:** Isadora Nicolielo de Souza, Maria Eduarda Pafetti Cristovam, Eduardo Leandro Moraes, Viviane Cristina Modesto, Naiane Antunes Alves Ribeiro, Vitória Almeida Moreira Girardi, Nelson Câmara de Souza Júnior, Aline Marchetti Silva Matos, Jussara Souza Salles, Camili Sardinha Gasparini, Wander Luís Barbosa Borges, Marcelo Andreotti

**Affiliations:** 1 Department of Plant Health, Rural Engineering and Soils, College of Engineering, São Paulo State University—UNESP-FEIS, Ilha Solteira 15385-088, SP, Brazil; isadora.nicolielo@unesp.br (I.N.d.S.); maria.pafetti@unesp.br (M.E.P.C.); el.moraes@unesp.br (E.L.M.); viviane.modesto@unesp.br (V.C.M.); na.ribeiro@unesp.br (N.A.A.R.); vitoria.almeida@unesp.br (V.A.M.G.); aline.marchetti@unesp.br (A.M.S.M.); jussara-souza.salles@unesp.br (J.S.S.); camili.gasparini@unesp.br (C.S.G.); marcelo.andreotti@unesp.br (M.A.); 2 Advanced Research Center for Rubber Tree and Agroforestry Systems, Agronomic Institute (IAC), Votuporanga 15505-970, SP, Brazil; wander.borges@sp.gov.br

**Keywords:** long-term systems, microbiology, microorganisms, residual effect, soil conditioner, systems management, tropical soils

## Abstract

Most tropical soils, as in the case of Brazil, are highly weathered, with low fertility, high acidity, and toxic aluminum, which limits crop management. Promoting root development is essential to overcome these constraints, and agricultural gypsum has shown positive effects in no-tillage systems. This study evaluated the residual effects of five gypsum rates in an integrated crop–livestock system, with or without inoculation of rotation grasses with *Azospirillum brasilense*, on crop productivity and soil fertility over 40 months. The experiment was conducted in a randomized block design with four replications in a 5 × 2 factorial scheme. Inoculated grasses increased yields of soybean, sorghum intercropped with Paiaguás grass, and black oat, whereas non-inoculated areas had the highest corn yield, likely due to hybrid metabolism. Gypsum had limited effects on crop yields, with lower doses performing slightly better. Inoculation improved soil fertility, increasing base sum, cation exchange capacity, and base saturation up to 0.60 m depth at 18 and 40 months. After 40 months, gypsum enhanced soil conditioning and increased calcium, sun of bases, and base saturation. Overall, inoculation with *Azospirillum brasilense* in rotation grasses under long-term no-tillage systems enhanced crop productivity and contributed to improved soil fertility.

## 1. Introduction

Most tropical soils—especially those in the Brazilian Cerrado biome—are highly weathered, with low fertility, high acidity (low pH), low base saturation (V%), and high concentrations of toxic aluminum (Al^3+^), among other characteristics that hinder their management [1]. One strategy to overcome these challenges is to provide conditions that favor better root development of crops, enabling greater soil profile exploration and, consequently, increasing water and nutrient uptake. The application of agricultural gypsum has been shown to improve root growth in areas under no-tillage systems (NTS) [2,3].

Liming carried out under NTS reduces soil acidity at the surface; however, its effects at deeper layers are limited, mainly due to the lack of soil incorporation and the low solubility of the amendment [4]. To improve subsurface conditions and complement the action of lime, gypsum has been used as a soil conditioner. Owing to its higher solubility, gypsum presents greater mobility in the soil profile, enriching deeper layers with nutrients and reducing Al^3+^ saturation, thereby stimulating deeper rooting and enhancing the development of crop root systems [5,6]. The application of gypsum has been associated with improvements in soil profile fertility, increasing nutrient availability at depth, particularly calcium (Ca), magnesium (Mg), and potassium (K) [7,8]. Its use as a subsurface conditioner can also enhance soil physical structure (macropores, micropores, and aggregates) through greater root development, thereby improving nutrient uptake and soil water content [9,10], in addition to being a sustainable by-product of phosphate fertilizer production.

In addition to chemical practices, such as the use of gypsum to improve subsurface soil conditions, biological approaches—such as the use of plant growth-promoting bacteria (PGPB), particularly those of the genus *Azospirillum brasilense*—emerge as environmentally friendly and cost-effective alternatives that are effective within integrated management strategies. PGPB act through the biosynthesis of phytohormones, secondary metabolites, and biological nitrogen fixation (BNF), inducing resistance to biotic and abiotic stresses while also enhancing the efficiency of mineral fertilizer use by increasing root volume [11,12]. Several studies have demonstrated the benefits of using *A. brasilense* as a PGPB. The mechanisms triggered by these bacteria are responsible for mitigating stresses and increasing productivity in different crops [13,14,15,16,17]. Well-managed soils under NTS tend to harbor a diverse microfauna capable of sustaining beneficial bacteria, which may supply the needs of the main crop even without new inoculations [18,19].

From the perspective of sustainable agriculture, management strategies become even more relevant considering that soil—the fundamental unit for global agricultural production—has been subjected to great pressure due to the evident population growth, resulting in physical, chemical, and biological degradation processes. Thus, the importance of strategic and adequate soil management is evident, aiming to make production progressively more sustainable [20]. In response to this degradation, the development and adoption of conservationist systems, such as NTS, integrated crop–livestock production systems (ICLSs), and the use of PGPB, have intensified. However, new questions arise regarding the management of amendments and fertilizers within these complex systems. For instance, there is still a lack of parameters for recommending the most efficient gypsum application rates, as well as uncertainties regarding the extent to which gypsum effects in such areas influence root development associated with PGPB use and, consequently, how these variables impact dry matter production and grain yield in an ICLS.

In this context, we hypothesized that there is an adequate gypsum application rate capable of efficiently conditioning the soil, resulting in more voluminous root systems which, in addition to exploring a greater soil volume, provide a better surface for PGPB colonization in these roots, thereby positively influencing crop development and productivity within the system. Furthermore, this effect may persist even when inoculations are carried out in rotation crops, ensuring greater sustainability and reducing costs. The aim of this study was to evaluate the residual effects of applying five rates of agricultural gypsum in an area under an ICLS, with or without *Azospirillum brasilense* inoculation in rotation grasses, as well as their effects on soil fertility and crop productivity over five years in a dystrophic Red Latosol (Typic Oxisol).

## 2. Results

### 2.1. Soil Fertility Results

Soil fertility was evaluated at two time points, 18 and 40 months after the application of agricultural gypsum. The data presented in Supplementary Materisla Table S1 and Table 1 show that, after 18 months of gypsum application, the mean values of soil chemical attributes in the 0–0.20 m layer did not present significant differences for gypsum rates or for the interaction (rates × inoculation). However, regarding the use of *A. brasilense*, the non-inoculated areas showed higher mean values for sulfur (S–SO_4_), Al, and potential acidity (H+Al). In contrast, the inoculated areas exhibited significantly higher means for pH, Ca, Mg, sum of bases (SB), cation exchange capacity (CEC), and V%. At 40 months (Table 1; Supplementary Materisla Table S2), in the same layer (0–0.20 m), for inoculation, the non-inoculated areas showed significantly higher means for organic matter (OM) and S–SO_4_, whereas the inoculated areas did not present significant differences in the means. Nonetheless, in terms of overall fertility, increments were observed in CEC, SB, and V%.

Regarding the 0.20–0.40 m layer (Table 1; Supplementary Materisla Table S3), at 18 months after gypsum application, for inoculation, the non-inoculated areas showed significantly higher mean values for S–SO_4_, organic matter, and Al. In contrast, the inoculated areas presented significant increases in Ca, Mg, sun of bases, CEC, and V%. At 40 months (Table 1; Supplementary Materisla Table S4), this effect persisted, with significant mean values for S–SO_4_, H+Al, and Al in the non-inoculated areas. In the inoculated areas, higher mean values were observed for pH, Ca, Mg, sun of bases, CEC, and V%. It is noteworthy that in both soil layers (0–0.20 m and 0.20–0.40 m), the results at 18 and 40 months after bacterial application exhibit a similar pattern, indicating that the use of *A. brasilense* contributes to soil fertility, particularly for attributes such as sun of bases, CEC, and V%. This suggests that the growth-promoting metabolism of these bacteria can influence soil structure, thereby affecting nutrient availability.

At 18 months (Table 1; Supplementary Materisla Table S5), in the 0.40–0.60 m layer, gypsum application rates had a significant effect on OM, with a maximum point observed at 3.1 t ha^−1^ of gypsum. Mean values of S–SO_4_ were significant in the non-inoculated areas, which may be associated with greater availability due to gypsum use and a lower proportion of roots for nutrient uptake at this depth. In contrast, the inoculated areas showed significant effects on phosphorus (P), organic matter, K, Ca, Mg, sun of bases, CEC, and V%. At 40 months (Table 1; Supplementary Materisla Table S6), for the 0.40–0.60 m layer, gypsum application rates significantly affected Ca, SB, and V%, with maximum points reached at 5.31, 4.40, and 5.71 t ha^−1^, respectively. Regarding inoculation, non-inoculated areas showed significant effects for S–SO_4_, H+Al, and Al. In the inoculated areas, mean values of pH, Ca, Mg, SB, and V% were significantly higher. Once again, these results indicate that the positive effects are maintained across cycles, demonstrating the viability of using PGPB.

### 2.2. Crop Yield and Productivity in the Field

For soybean in the three cropping cycles evaluated, considering the inoculation factor, there was a significant difference in grain yield (GY 1) between the periods 10/2019–03/2020 (GY 2) and 11/2020–03/2021 (GY 3) (*p* ≤ 0.05) (Table 2), in which treatments with preceding grasses inoculated with *A. brasilense* provided the highest grain yields. With inoculation, grain yield in the second soybean cycle (GY 2) increased by approximately 14%, while grain yield (GY 3) showed an increase of about 17%. This demonstrates the beneficial effect of using PGPB of the genus *Azospirillum*, which are capable of promoting yield gains through their positive effects on soil profile exploration, proper nutritional balance, and improved metabolism. Furthermore, based on the data in Table 1, it is possible to observe that grains yield (GY 3) showed significance for the interaction (*p* ≤ 0.05).

**Figure 1:**
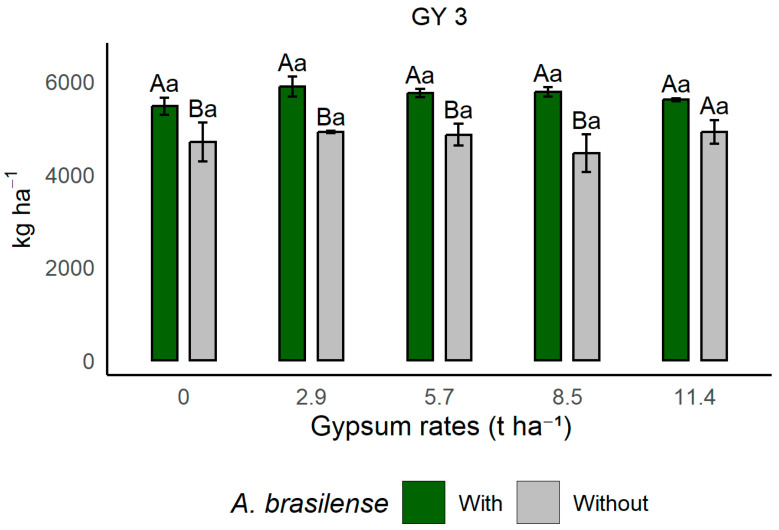
Effects of gypsum rate and co-inoculation with PGPB on soybean grain yield during the period 11/2020–02/2021 (GY3) in Selvíria, Mato Grosso do Sul State. Means followed by different uppercase letters, within treatments with or without inoculation with *A. brasilense*, and by different lowercase letters, within the different gypsum doses, differ from each other according to Tukey’s test at the 5% probability level. With: areas where *A. brasilense* was inoculated in the rotation crop; Without: areas where *A. brasilense* was not inoculated. GY 3: Grain yield in the third soybean cycle—11/2020 to 02/2021.

The interaction data for grain yield (GY 3) (Figure 1) show that, regardless of gypsum application rates, all treatments inoculated with *A. brasilense* presented higher means compared to the non-inoculated treatment, with the exception of the treatment with 11.4 t ha^−1^ of gypsum, which showed a mean equal to that of the inoculated treatment. It is noteworthy that the control treatment (without gypsum application) but co-inoculated with *A. brasilense* presented a mean equal to the other treatments with gypsum application. This occurred due to the history of the experimental area under long-term no-tillage system, which balanced soil fertility. However, it is important to emphasize the growth-promotion mechanism of the bacteria, capable of increasing soybean grain yield, thereby supporting the use of these PGPB in this crop.

The analysis of variance for shoot dry matter (SDM), sorghum grain yield (SGY), and forage dry matter (DM) (Table 3) showed significant means for the inoculation factor according to Tukey’s test (*p* ≤ 0.05). Inoculation with *A. brasilense* increased sorghum SDM production by 45%, while its SGY was enhanced by approximately 16%. Forage production was also positively affected by PGPB inoculation, with a 17% increase in its final DM volume.

**Figure 7:**
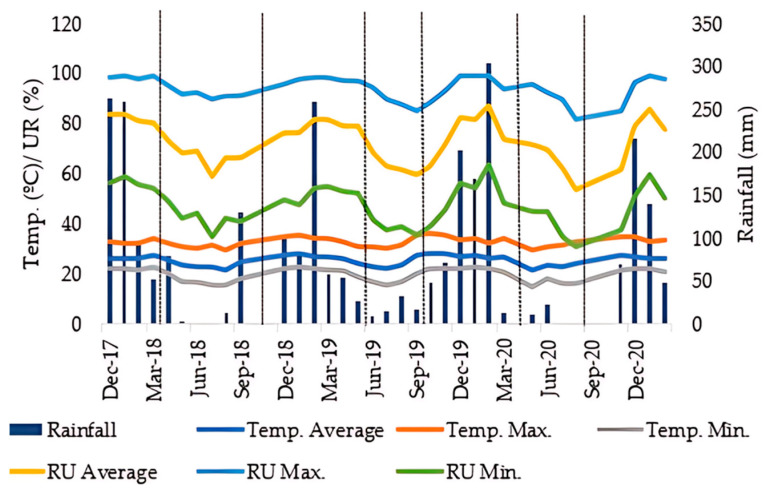
Monthly climatic data of average temperature and rainfall from November 2017 to March 2021, Selvíria, State of Mato Grosso do Sul, Brazil.

It is noteworthy that soybean grain yield (GY) (Table 2) and the means for sorghum SDM, SGY, and forage DM (Table 2) did not show significant effects for gypsum application. This can be explained by the fact that the analyzed period had lower rainfall incidence, which affected soil water availability (Figure 7), thereby influencing gypsum solubility.

The means for maize grain yield (MGY) and Massai grass dry matter (MDM) (Table 4) showed significant differences according to Tukey’s test (*p* ≤ 0.05). Maize MGY and Massai grass MDM presented significant means for the interaction between gypsum rates and inoculation.

**Figure 2:**
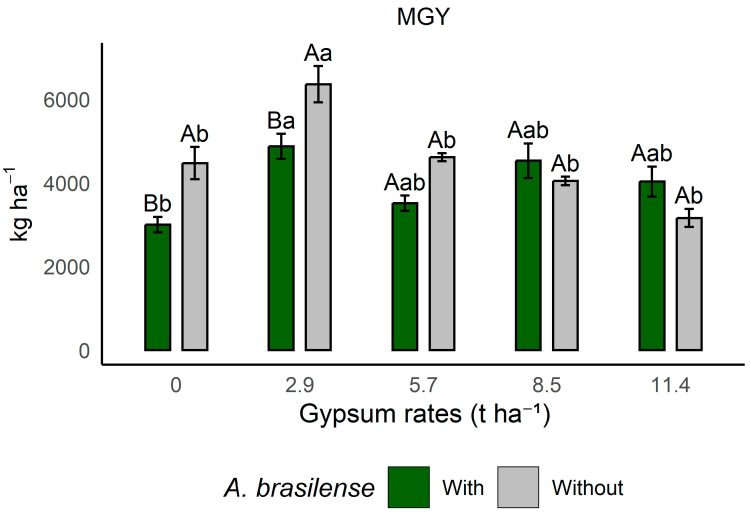
Effects of gypsum rate and co-inoculation with PGPB on maize grain yield during the period 12/2018–05/2019 (MGY) in Selvíria, Mato Grosso do Sul State, Brazil. Means followed by different uppercase letters, within treatments with or without inoculation with *A. brasilense*, and by different lowercase letters, within the different gypsum doses, differ from each other according to Tukey’s test at the 5% probability level. With: areas where *A. brasilense* was inoculated in the rotation crop; Without: areas where *A. brasilense* was not inoculated. MGY: Maize grain yield.

For the interaction, maize grain yield (MGY) (Figure 2) shows that the highest mean production was observed in the treatment with 2.9 t ha^−1^ of gypsum without PGPB inoculation, a result 30% higher than the control. The higher gypsum rates experienced solubilization problems due to water deficit conditions (Figure 2) during the crop season. The lower yield in the inoculated areas may be related to the physiology of the crop as a C_4_ plant.

**Figure 3:**
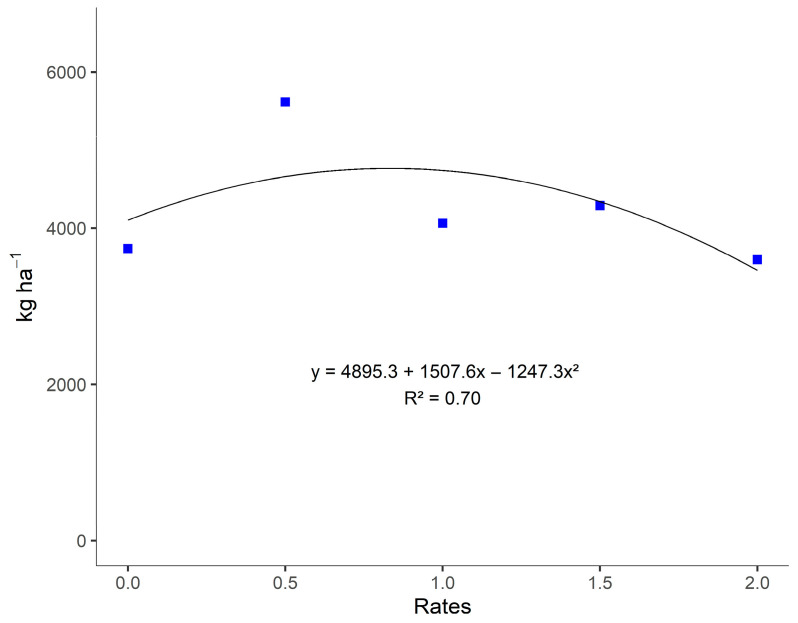
Regression of the effects of gypsum application rates without inoculation with *A. brasilense* on corn grain yield (MGY) from December 2018 to May 2019, in Selvíria, Mato Grosso do Sul State, Brazil. **Note:** Control dose (0.0); half of the standard dose = 2.9 kg ha^−1^ (0.5); standard dose = 5.7 kg ha^−1^ (1.0); standard dose + half = 8.5 kg ha^−1^ (1.5); double the recommended dose = 11.4 kg ha^−1^ (2.0).

In the regression analysis for the breakdown of gypsum rates, a quadratic adjustment was observed for the treatments without inoculation regarding maize grain yield (MGY) during the period from December 2018 to May 2019 (Figure 3). When analyzing the coefficient of determination (R^2^), we found that the proportion of variance was fitted at 0.6954, with an optimal dose of 0.6 t ha^−1^, resulting in a grain yield of 5351 kg ha^−1^. In the case of maize, it is possible to observe that the supply of Ca and S provided by gypsum at lower doses was more decisive in ensuring a good grain yield.

**Figure 4:**
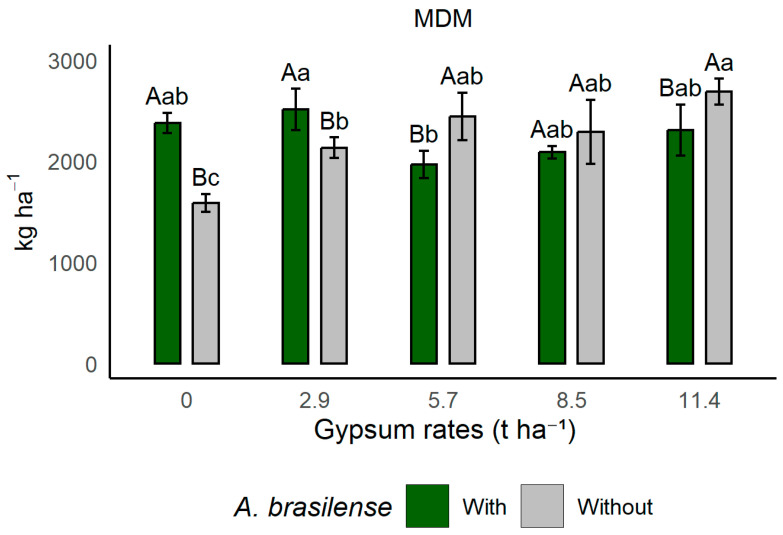
Effects of gypsum application rates and co-inoculation with PGPB on dry matter yield of Massai grass (MDM) from December 2018 to May 2019, in Selvíria, Mato Grosso do Sul State, Brazil. Means followed by different uppercase letters, within treatments with or without inoculation with *A. brasilense*, and by different lowercase letters, within the different gypsum doses, differ from each other according to Tukey’s test at the 5% probability level. With: areas where *A. brasilense* was inoculated in the rotation crop; Without: areas where *A. brasilense* was not inoculated. MDM: Massai grass dry matter production.

In the interaction between gypsum application rates and inoculation for dry matter yield of Massai grass (MDM) from December 2018 to May 2019 (Figure 4), residual gypsum rates of 0 and 2.9 t ha^−1^ resulted in higher production in inoculated areas, whereas in non-inoculated areas this effect was significant only at the higher residual gypsum rates (5.7 and 11.4 t ha^−1^). These results indicate that lower gypsum rates may be more beneficial when combined with *A. brasilense* inoculation, due to its hormonal effect and colony behavior. In contrast, in non-inoculated areas, without the bacterial hormonal effect, higher gypsum rates improved subsoil fertility and provided better conditions for root growth through the supply of Ca and S. However, higher gypsum rates may cause instability in the mechanisms of PGPB, reducing their efficiency, as observed in this study.

**Figure 5:**
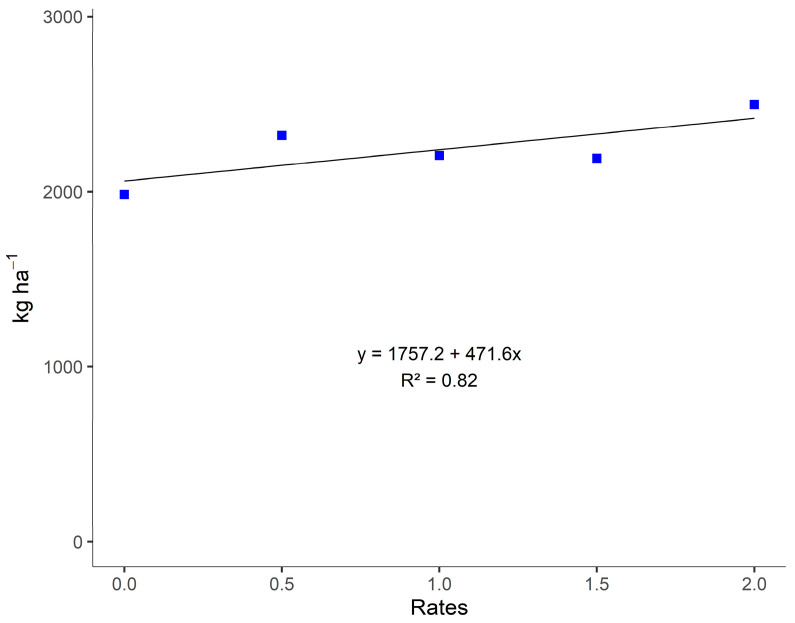
Regression of the effects of gypsum application rates without inoculation with *A. brasilense* on dry matter yield of Massai grass (MDM) from December 2018 to May 2019, in Selvíria, Mato Grosso do Sul State, Brazil. **Note:** Control dose (0.0); half of the standard dose = 2.9 kg ha^−1^ (0.5); standard dose = 5.7 kg ha^−1^ (1.0); standard dose + half = 8.5 kg ha^−1^ (1.5); double the recommended dose = 11.4 kg ha^−1^ (2.0).

Analyzing the regression for gypsum application rates, a linear increasing trend was observed only in the non-inoculated areas (Figure 5), indicating that dry matter production increased with higher gypsum rates. Since agricultural gypsum provides Ca and S as nutrients, which are essential for forage grasses, it can be stated that the greater availability of these elements broadly influences the dry matter yield of Massai grass.

In black oat, gypsum did not have a significant effect on means in either of the evaluated cycles. Evaluating the effects of inoculation, significant differences in dry matter yield were observed from June 2019 to September 2019 (DMBO 1) in inoculated areas (Table 5). It should be noted that high temperatures during this growth period contributed to the low biomass production, as black oat performs better under mild conditions. Although no differences were detected using Tukey’s test (*p* ≤ 0.05), inoculated areas in DMBO 2 (May 2020 to August 2020) produced 755 kg ha^−1^ more biomass than non-inoculated areas. This suggests that black oat responds positively to the use of PGPB, which can help improve biomass production even under temperature fluctuations, as observed in this study.

## 3. Discussion

### 3.1. Soil Fertility

The use of *A. brasilense* in rotational grasses enhances soil fertility, particularly in parameters such as SB, CEC, and V% (Table 1; Supplementary Materisla Tables S1–S6), due to the metabolic effects provided by PGPB activity. Bacteria of this genus are capable of producing phytohormones, such as auxins, gibberellins, and cytokinins, which stimulate deeper root growth, in addition to producing biofilms that aid in nutrient protection and availability [21,22,23]. Greater soil exploration can lead to important changes in the soil profile, increasing overall fertility [24]. Inoculation of PGPB in grasses offers significant benefits related to the interaction between these bacteria and the plant. Grasses possess a large fibrous root system capable of deep soil exploration, which favors bacterial–root contact and improves the interaction between organisms [25,26]. In this interaction, grass roots often exude sugars, organic acids, and amino acids, which can serve as an energy source for microbial colonies, in turn significantly affecting soil fertility and nutrient availability [14,18].

The results for soil fertility in this study confirm that the use of PGPB of the genus *A. brasilense* contributes to soil fertility throughout the cycles of different crops. A strong correlation between bacterial application and increased soil fertility (SB, CEC, and V%) is evident (Table 1; Supplementary Materisla Tables S1–S6), including improvements in deeper soil layers over the long term, explained by the promotion of root growth (Table 1; Supplementary Materisla Table S6) [18,25]. In this study, in consolidated no-tillage systems (NTS), lower gypsum rates were associated with the best fertility results. This may be related to soil management practices that improve aggregate structure, increase organic matter content, and enhance biological activity, thereby reducing the need for gypsum application [27].

The use of agricultural gypsum should not be dismissed in tropical agriculture, given its effect as a soil conditioner in deeper layers. This was observed in the increases in Ca, SB, and V% 40 months after application (Table 1; Supplementary Materisla Table S6). Additionally, gypsum use contributes to deeper root exploration in the soil profile, which is particularly important in tropical regions that can be strongly affected by temperature fluctuations [28,29]. Its use is associated with increased productivity across different crops, as well as improvements in soil physical and chemical conditions, as presented in different studies [30,31,32]. The long-term effect promotes sustainable benefits for agricultural practice, reducing reliance on fertilizers and consequently production costs. The interaction between gypsum rates and the use of *A. brasilense* did not result in significant differences in means in this study. It is noteworthy that long-term management in consolidated NTS soils may show less pronounced responses to management practices. However, the isolated effects of PGPB inoculation and gypsum application demonstrate that, even in consolidated NTS soils, these strategies can significantly improve soil fertility parameters, representing sustainable gains in agricultural productivity.

### 3.2. Crop Productivity

Soybean shows positive responses to co-inoculation with *B. japonicum* and *A. brasilense*, where growth-promoting mechanisms—particularly root development and increased root hair density—are important for enhancing biological nitrogen fixation (BNF), which contributes to increased productivity [33,34,35,36]. By improving these mechanisms, plants expand their capacity to explore the soil profile, facilitating access to groundwater, directly influencing plant water content, enhancing physiological metabolism, mitigating field stresses, and ensuring higher yield [37,38,39,40]. Due to these mechanisms, PGPB can also benefit nodule formation in legumes through increased root volume [41,42]. These mechanisms influenced by PGPB positively affect the productivity of biomass in rotational crops, as observed in sorghum, Paiaguás grass, and black oat (Table 3, Table 4 and Table 5) and reported in other studies [43,44,45].

The use of *A. brasilense* positively affects plant metabolism, as bacteria of this genus can help regulate auxins, indolic compounds, gibberellins, and cytokinins [40,44,45]. In general, these phytohormones act on plant development, promoting cell elongation and growth, as well as improving nutritional balance [35,41,46]. Soybean responds well to *A. brasilense* inoculation, and this study confirms that even inoculation in preceding grasses can maintain an efficient microfauna in well-managed soils. ICLSs use soil cover as a protective measure, and over time organic matter contributes more significantly, being one of the factors associated with increased soybean productivity, as observed in this study and in others [47,48]. Therefore, soils with low nutrient levels and low OM limit grain yield.

The absence of response to inoculation during the period from November 2017 to March 2018 (GY1) (Table 2) may be related to the low organic matter content, which can affect the maintenance of the colony in the soil. Another factor to consider is temperature fluctuation (Figure 7), especially in tropical areas. *A. brasilense* bacteria are Gram-negative and highly sensitive to thermal fluctuations, with high temperatures potentially inhibiting their growth and causing cell death. In this context, OM acts as a buffer, helping to maintain active colonies in the soil and minimizing microbial mortality, what has been observed in different studies on long-term NTS systems [41,42,47,48].

When evaluating the residual effect of gypsum rates applied in 2017/18 and 2019/20 (Table 1), no effects on soybean yield were observed, as also reported by several authors who did not find yield increases with agricultural gypsum application [49,50,51]. This effect has been attributed to adequate fertility in the topsoil and the climatic conditions during the soybean growth cycle (Figure 7). Studies indicate that soybean does not respond to gypsum application or that the sulfur supplied through OM mineralization is sufficient to meet crop requirements [30,50,52], which is confirmed by the results observed in the control treatment without gypsum addition (rate 0) for soybean in GY 3 (2020/21) (Figure 1) in this study.

Another factor explaining the lack of soybean response to gypsum application is that root growth was not limited by low aluminum (Al) levels in the soil [53]. However, in more acidic soils, gypsum application increased soybean yield by 11.4% with lime addition and by 11.3% without lime [54]. It was also found that high gypsum rates did not significantly contribute to the yield of soybean, black oat, or Paiaguás grass. This may be related to nutrient leaching caused by excessive gypsum application, and the increase in Ca may lead to greater competition with Mg for the same absorption sites, negatively affecting nutrient balance and potentially compromising photosynthetic rates [30,55,56].

For maize (Figure 2), the descriptive statistics confirmed, based on the interaction results, that the application of 2.9 t ha^−1^ of gypsum without inoculation was responsible for the highest crop yield. The lower productivity observed in inoculated areas may be associated with the low response of the crop to inoculation, since the efficiency of this process varies among species [57]. In addition, the maize varieties available on the market differ in their responsiveness to PGPB, with some genotypes being more responsive than others. Typically, high-performance hybrids tend not to show significant gains with inoculation [58,59]. Temperature fluctuations and water deficit may also have compromised the maintenance of a viable and active bacterial colony in the system (Figure 2). Overall, maize response is strongly influenced by the system’s history, with important contributions from organic matter and balanced soil fertility in consolidated no-tillage (NT) systems. Regarding the use of agricultural gypsum, previous studies confirm that gypsum increases maize yield by improving root development and soil profile conditions, providing better nutrient and water balance for the crop [30]. Maize can develop a root volume that extends up to 0.6 m in the surface soil layer, and in well-managed soils, roots can reach depths of up to 2 m [60,61].

For Massai grass (Figure 4), the interaction results indicated that gypsum rates of 0 and 2.9 t ha^−1^ with inoculation did not differ significantly. This outcome is related to proper soil management and the metabolic effects promoted by *A. brasilense*. These findings suggest that, with bacterial inoculation, lower gypsum rates may be more efficient than higher ones. The greater dry matter accumulation of Massai grass observed at higher residual gypsum rates is explained by the greater subsoil correction and higher S concentrations, since this nutrient is essential for protein and chlorophyll synthesis, thereby stimulating vigorous plant growth. On the other hand, sulfur deficiency reduces protein and sugar synthesis, consequently decreasing dry matter production in forages [62].

This long-term study confirms that the residual effect of *A. brasilense* in soils managed under long-term NTS systems is real and effective for crops. Throughout the study, it was observed that the gypsum rates applied had a limited contribution to the productivity of different crops over the cycles. Overall, what this study shows is that managing tropical soils under long-term NTS is the most effective method to ensure system maintenance and achieve good productivity levels. Over the years, due to proper soil management, a positive trend in soil fertility can be observed even in the control areas. This means that maintaining the pillars of NTS, with crop rotation, organic matter maintenance, and minimal soil disturbance, is capable of sustaining agricultural crops. This has been evidenced by various studies confirming the need to maintain a consolidated no-tillage system to reduce costs, improve soil fertility, and increase profitability [63,64,65].

Agricultural gypsum can help in building the soil profile, making fertility more balanced in deeper layers [63,64]. This study found that it is not necessary to apply excessive rates of gypsum in long-term NTS, where the system itself is able to improve soil fertility over the years. Gypsum is relatively inexpensive, generally costing no more than USD 6.00 ha^−1^, and can be used in a balanced manner. In the case of using *A. brasilense*, its low cost, approximately USD 4.00 ha^−1^, favors and makes its use feasible in tropical countries. The value of a 60 kg sack of soybean is around USD 23.00, and in this study an increase of about 14–16 sacks ha^−1^ was observed with the use of *A. brasilense*. The gains from using the bacterium demonstrate that its application can be important to ensure high production and profitability. This study confirms that maintaining soil under long-term NTS is one of the most sustainable practices benefiting soil systems in tropical countries. Long-term studies can demonstrate soil behavior throughout agricultural cycles and elucidate important management techniques for this type of system. The findings of this study indicate that the maintenance of soil organic matter (OM) can influence overall soil fertility and crop productivity [66,67]. With the benefits provided by long-term NTS, studies focusing on new management practices become essential, aiming to compare or propose techniques that can contribute to the maintenance of this system. The results obtained in the control treatment confirm that good agricultural practices are essential for sustaining agriculture, ensuring a system capable of supporting itself.

## 4. Materials and Methods

### 4.1. Characterization of the Experimental Area

**Figure 6:**
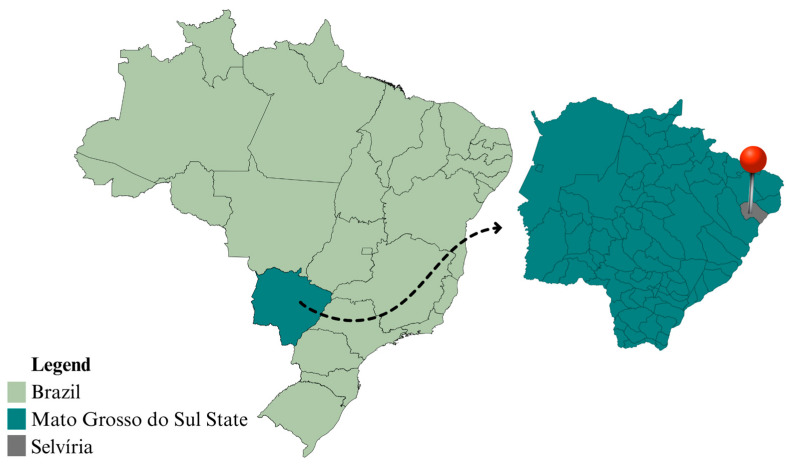
Map of the experiment site in a Cerrado area under a long-term no-tillage system, Selvíria, State of Mato Grosso do Sul, Brazil, 2018/2021.

The research was developed in the municipality of Selvíria, state of Mato Grosso do Sul, Brazil (20°20′05″ S and 51°24′26″ W, altitude of 335 m), at the Teaching, Research and Extension Farm in the Plant Production Sector of São Paulo State University, Faculty of Engineering of Ilha Solteira (Figure 6).

The climate of the experimental area is characterized as Aw according to the Köppen classification [68]: humid tropical with dry winters and rainy seasons during the summer. The climatic conditions during the experiment are shown in Figure 7.

According to the Brazilian Soil Classification System [69], the soil at the research site is a typical clayey dystrophic RED LATOSOL (typic Oxisol) with 520 g kg^−1^ clay.

Before the experiment was performed, 30 simple soil samples were collected from the 0–0.20 and 0.20–0.40 m layers (August 2017) to compose a composite sample, whose chemical attribute results [70] are shown in Table 6.

### 4.2. Experimental Design and Treatment Formulation

The experimental design used was randomized blocks, with four replications, in a 5 × 2 factorial scheme, with five doses of gypsum, with or without inoculation with the bacterium *A. brasilense* in the grasses in rotation. Each experimental unit was 9 m wide and 7 m long, totaling 63 m2 per plot, and the total experimental area was 2520 m^2^.

The gypsum doses used were the control (0 t ha^−1^), ½ dose (2.9 t ha^−1^), standard dose (5.7 t ha^−1^), and 1.5 dose (8.5 t ha^−1^), and double the dose (11.4 t ha^−1^), which were calculated according to the methodology of Caires and Guimarães (2018) [71] via the following formula: $NG=\left[\left(0.6\times CECef\right)-Ca\right]\times 6.4$, where “NG” represents the need for gypsum, given in kg ha^−1^; “CECef” represents the adequate cation exchange capacity; and “Ca” corresponds to the calcium content, provided in cmolc dm^−3^ in the 0.20 to 0.40 m layer of the soil. In several countries, agricultural gypsum is still little used, although there is evidence that different application rates can generate distinct responses in the soil. In this study, the rates were defined to evaluate the effect of the standard dose, considering its reduction and increase, on soil dynamics and crop productivity over multiple growing cycles. The use of gypsum is economically viable due to its low cost, generally less than USD 6.0 per ton. However, transportation to the farm can raise the final price, making it essential to define an appropriate rate that justifies its application and contributes to reducing production costs.

### 4.3. Experimental Management and Handling Throughout the Crop Cycles

On 11 June 2017, gypsum was applied to the experimental plots according to each treatment, being broadcast on the soil surface without incorporation. The area had a history of annual cropping under no-tillage system (NTS) for eight years, and the last four growing seasons (2017/2018, 2018/2019, 2019/2020, and 2020/2021) were the focus of the present research (Figure 3).

From November 2017 to March 2018, soybean (cv. BRS Potência, EMBRAPA, Londrina, Brazil) was cultivated, sown on 9 November 2017, at a spacing of 0.45 m between rows and a seeding density of 350,000 plants ha^−1^. Seeds were inoculated with *Bradyrhizobium japonicum* (strain SEMIA 5079—5 × 10^9^ CFU g^−1^, at a rate of 100 g per 40 kg of seeds). The residual effect of inoculation with *Azospirillum brasilense* (strains AbV5 and AbV6—9 × 10^8^ CFU mL^−1^, applied at 200 mL per 20 kg of seeds) from the previous crop was also assessed. Sowing fertilization consisted of 240 kg ha^−1^ of the 08-28-16 formulation. Harvest was carried out on 25 March 2018, when grain yield was determined.

From April to September 2018, grain sorghum (cv. Ranchero, A9904, Semeali, Birigui, Brazil) was intercropped with *Urochloa brizantha* cv. Paiaguás. Sowing was performed on 9 April 2018, at a row spacing of 0.45 m and a population of 222,220 sorghum plants ha^−1^. Seeds were either inoculated or not, according to treatments, with *A. brasilense* (strains AbV5 and AbV6—9 × 10^8^ CFU mL^−1^, at a rate of 150 mL per 20 kg of seeds). For Paiaguás palisade grass, 6 kg ha^−1^ of seeds with a cultural value (CV) of 78% were sown between the sorghum rows. Base fertilization consisted of 330 kg ha^−1^ of the 08-28-16 formulation. Sorghum was harvested for silage on 25 July 2018, at grain physiological maturity (approximately 70% DM in the grains). Simultaneously, Paiaguás grass was left fallow and desiccated with glyphosate (1440 g ha^−1^ a.i.) on 30 September 2018, to form straw. At this stage, dry matter yield was evaluated by collecting two 1 m^2^ samples per plot using a metal frame (1.0 × 1.0 m).

From December 2018 to May 2019, maize (cv. Agroceres 7908 VT PRO 2, Agroceres, Rio Claro, Brazil) was intercropped with *Megathyrsus maximus* cv. Massai (62% CV). Sowing took place on 4 December 2018, with 0.45 m row spacing and a plant population of 73,333 maize plants ha^−1^. The residual effect of inoculation with *A. brasilense* (strains AbV5 and AbV6—9 × 10^8^ CFU mL^−1^) from the previous crop was evaluated. For Massai grass, a seeding rate of 5 kg ha^−1^ was adopted. Fertilization consisted of 350 kg ha^−1^ of the 08-28-16 formulation at sowing and 500 kg ha^−1^ of 20-00-20 as topdressing on 1 April 2019. Maize was harvested on 18 March 2019, when grain yield was determined. Massai grass was left fallow and later desiccated with glyphosate (1440 g ha^−1^ a.i.) on 13 May 2019, when dry matter yield was assessed by collecting two 1 m^2^ samples per plot using a metal frame.

From June to September 2019, black oat (*Avena strigosa*) cv. EMBRAPA 29 (EMBRAPA, Londrina, Brazil) was cultivated, sown on 6 May 2019, with 0.17 m row spacing and a density of 60 seeds m^−1^. Seeds were either inoculated or not with *A. brasilense* (strains AbV5 and AbV6—9 × 10^8^ CFU mL^−1^, at a rate of 150 mL per 25 kg of seeds), depending on treatments. For topdressing, 50 kg ha^−1^ of N (ammonium sulfate) was applied. Desiccation with glyphosate (1440 g ha^−1^ a.i.) and plant sampling for dry matter yield determination were carried out on 21 September 2019, following the same methodology used for forage sampling.

From October 2019 to March 2020, soybean (cv. TMG 7063 IPRO 2, TMG, Cambé, Brazil) was cultivated, sown on 5 October 2019, at a row spacing of 0.45 m and a seeding density of 350,000 plants ha^−1^. Seeds were inoculated with *B. japonicum* (strain SEMIA 5079—5 × 10^9^ CFU g^−1^, at a rate of 100 g per 40 kg of seeds), and the residual effect of *A. brasilense* (strains AbV5 and AbV6—9 × 10^8^ CFU mL^−1^, applied at 200 mL per 20 kg of seeds) was also evaluated. Fertilization at sowing consisted of 414 kg ha^−1^ of the 02-20-20 formulation. Harvest was carried out on 4 March 2020, when grain yield was determined.

Black oat cv. EMBRAPA 29 was grown from May to August 2020, sown on 30 May 2020, at 0.17 m row spacing and a density of 60 seeds m^−1^. Seeds were either inoculated or not with *A. brasilense* (strains AbV5 and AbV6—9 × 10^8^ CFU mL^−1^, at a rate of 150 mL per 25 kg of seeds). For topdressing, 50 kg ha^−1^ of N (ammonium sulfate) was applied. On 2 August 2020, the crop was desiccated with glyphosate (1440 g ha^−1^ a.i.) to form straw. At this time, dry matter yield was assessed using the same methodology applied for oats in 2019.

**Figure 8:**
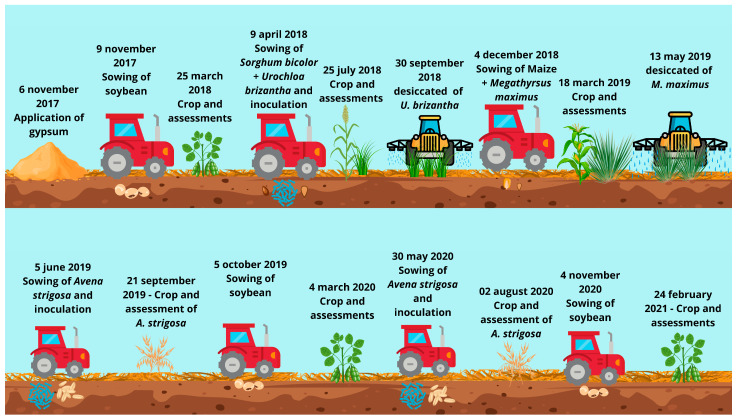
Crop Management in a Long-Term No-Tillage System in Cerrado Soil, Selvíria, Mato Grosso do Sul State, Brazil (2018–2021).

From November 2020 to February 2021, soybean (cv. TMG 7063 IPRO 2) was cultivated, sown on 4 November 2020, at a row spacing of 0.45 m and a seeding density of 326,663 plants ha^−1^. Seeds were inoculated with *B. japonicum* (strain SEMIA 5079—5 × 10^9^ CFU g^−1^, at a rate of 100 g per 40 kg of seeds), and the residual effect of *A. brasilense* (strains AbV5 and AbV6—9 × 10^8^ CFU mL^−1^, applied at 200 mL per 20 kg of seeds) was also evaluated. Sowing fertilization consisted of 414 kg ha^−1^ of the 02-20-20 formulation. Harvest was performed on 24 February 2021, when grain yield was analyzed (Figure 8).

The assessments conducted included total grain yield, in which four 4 m long rows were harvested, threshed, and weighed, and the values were extrapolated to kg ha^−1^. For the rotational grasses, samples were collected from the central square (3 samples per plot), weighed, and taken to an oven for determination of dry matter (DM), and subsequently the values were extrapolated to kg ha^−1^. For soil fertility analysis, 10 sampling points were collected at each depth; the samples were then mixed to obtain a composite sample and taken to the laboratory for fertility evaluation (Figure 8) [65].

### 4.4. Statistical Analyses

Data normality was tested using the Shapiro–Wilk test. The results were subjected to analysis of variance (ANOVA) via the F-test (*p* < 0.05). Tukey’s test was used to evaluate the effect of *Azospirillum* inoculation, while polynomial regression analysis was applied for gypsum doses. Statistical analyses and interaction plots were performed using R software version 4.5.1 [72]. The packages “ExpDes.pt”, “ggplot2”, “patchwork”, and “ggpattern” were used.

## 5. Conclusions

The use of *A. brasilense* improves soil fertility at greater depths (up to 0.60 m), positively influencing SB, CEC, and base saturation (V%). The application of agricultural gypsum after 40 months is able to condition the soil and affect parameters such as Ca content, SB, and V%. The use of *A. brasilense* in rotation grasses can provide beneficial effects and influence the yield of soybean, Massai and paiaguás grasses, sorghum, and black oat. The effect of *A. brasilense* inoculated in rotation grasses under long-term no-tillage systems (NTS) can be transferred to the main crops. High doses of agricultural gypsum showed the same results as standard doses, indicating that increasing gypsum rates does not influence crop yield. Maize responds positively to lower doses of agricultural gypsum in well-managed areas. Well-managed soils under consolidated NTS, both in terms of crop yield and soil fertility, tend to show increased natural fertility, indicating a lower need for inputs and reducing production costs.

## Figures and Tables

**Table 1:** Values of sum of bases (SB), cation exchange capacity (CEC), and base saturation (V%), in areas with or without inoculation with A. brasilense and with the use of agricultural gypsum, at soil depths of 0.00–0.20 m, 0.20–0.40 m, and 0.40–0.60 m, at 18 and 40 months after gypsum application in the municipality of Selvíria, Mato Grosso do Sul State, Brazil.

	18 Months			40 Months			18 Months			40 Months			18 Months			40 Months		
	0.00–0.20 m						0.20–0.40 m						0.40–0.60 m					
TRET	SB	CEC	V	SB	CEC	V	SB	CEC	V	SB	CEC	V	SB	CEC	V	SB	CEC	V
	mmolc dm^−3^		%	mmolc dm^−3^		%	mmolc dm^−3^		%	mmolc dm^−3^		%	mmolc dm^−3^		%	mmolc dm^−3^		%
without	25.8 b	67.6 b	39.8 b	55.3	93.8	56.6	22.7 b	52.7 b	42.9 b	23.1 b	60.0 b	38.7 b	18.7 b	41.2 b	45.1 b	21.9 b	54.1	40.6 b
with	41.9 a	76.0 a	53.2 a	64.8	99.8	61.8	31.3 a	58.3 a	53.3 a	33.0 a	64.3 a	50.7 a	26.3 a	48.1 a	53.6 a	28.7 a	55.8	51.0 a
0	29.0	71.1	38.9	71.4	107.8	61.4	21.4	52.4	41.0	26.7	58.2	41.9	17.9	40.6	43.6	22.6 ^1^	52.4	43.1 ^2^
2.9	33.3	66.7	43.4	61.8	96.1	63.4	25.5	50.7	50.1	27.2	62.8	46.0	22.4	43.1	50.9	24.4	52.4	46.0
5.7	37.3	74.8	49.0	58.8	96.0	58.0	30.5	60.5	50.1	27.7	65.0	42.8	25.1	48.3	51.5	25.9	57.4	45.0
8.5	30.6	65.0	43.4	46.2	89.3	51.0	26.6	54.6	47.6	28.8	63.1	46.9	22.9	45.4	49.9	27.1	56.8	47.3
11.4	31.9	81.5	58.9	62.1	95.0	62.1	31.0	59.3	51.6	29.8	61.9	45.9	24.1	45.9	50.9	26.5	55.6	47.5
Pr > Fc (I)	0.001 *	0.001 *	0.02 *	0.31 ^ns^	0.35 ^ns^	0.36 ^ns^	0.01 *	0.02 *	0.001 *	0.0001 *	0.04 *	0.001 *	0.0001 *	0.001 *	0.001 *	0.01 *	0.35 ^ns^	0.001 *
Pr > Fc (G)	0.26 ^ns^	0.20 ^ns^	0.33 ^ns^	0.56 ^ns^	0.48 ^ns^	0.65 ^ns^	0.07 ^ns^	0.41 ^ns^	0.18 ^ns^	0.40 ^ns^	0.32 ^ns^	0.53 ^ns^	0.30 ^ns^	0.38 ^ns^	0.40 ^ns^	0.26 ^ns^	0.28 ^ns^	0.35 ^ns^
Pr > Fc (I × G)	0.42 ^ns^	0.33 ^ns^	0.68 ^ns^	0.26 ^ns^	0.22 ^ns^	0.31 ^ns^	0.08 ^ns^	0.43 ^ns^	0.39 ^ns^	0.53 ^ns^	0.19 ^ns^	0.66 ^ns^	0.25 ^ns^	0.52 ^ns^	0.88 ^ns^	0.38 ^ns^	0.48 ^ns^	0.56 ^ns^
CV(%)	11.9	12.7	23.0	24.7	20.8	24.6	24.9	13.1	20.1	21.9	10.2	15.71	26.9	15.9	14.1	17.2	10.6	10.3

**Note:** P-resin: phosphorus; S-SO_4_: sulfur; OM: organic matter; pH: hydrogen potential; K: potassium; Ca: calcium; Mg: magnesium; H+Al: potential acidity; Al: aluminum; SB: sum of bases; CEC: cation exchange capacity; V: base saturation; TRET: treatments; CV (%): coefficient of variation. Means followed by different letters in the column for different treatments differ according to Tukey’s test at 5% probability. *: significant at 5% probability; ns: not significant; ^1^ $y=22.57\;+2.11x-\;0.24x^{2}\;(R^{2}=0.99)$; ^2^ $y=\;43.57\;+\;1.37x\;-\;0.12x^{2}\;(R^{2}=0.82)$. The results of all soil analysis parameters are provided in the Supplementary Material of this article.

**Table 2:** Soybean grain productivity (GY) from 11/2017 to 03/2018 (GY 1), 10/2019 to 03/2020 (GY 2), and 11/2020 to 02/2021 (GY 3) for the three soybean crops in the system due to the effects of inoculation by *A. brasilense* and residual gypsum after 4, 23, and 36 months of application, respectively.

Treatments	GY 1	GY 2	GY 3
	kg ha^−1^		
Gypsum rates (G) (t ha^−1^)			
0	3728	3696	5170
2.9 5.7	3836 3902	3756 3883	5401 5302
8.5	3829	3458	5113
11.4	3768	3921	5259
Inoculation (I)			
with	3790	4030 a	5733
without	3835	3455 b	4765
Pr > Fc (G)	0.849 ^ns^	0.714 ^ns^	0.840 ^ns^
Pr > Fc (I)	0.663 ^ns^	0.011 *	0.001 *
Pr > Fc (G × I)	0.837 ^ns^	0.823 ^ns^	0.001 *
DMS	211	462	348
CV (%)	8.54	19.12	10.22

Means followed by different letters in the column for different treatments differ according to Tukey’s test at 5% probability. *: significant at 5% probability ^ns^ not significant. Where: CV is the coefficient of variation, and DMS is the least significant difference. GY 1: Grain yield in the first soybean cycle—11/2017 to 03/2018; GY 2: Grain yield in the second soybean cycle—10/2019 to 03/2020; GY 3: Grain yield in the third soybean cycle—11/2020 to 02/2021.

**Table 3:** Shoot dry matter production of sorghum (SDM), sorghum grain yield (SGY), and forage dry matter production (DM) during the sorghum–Paiaguás grass intercropping system, from 04/2018 to 09/2018, evaluated as a function of the effects of *A. brasilense* inoculation and residual gypsum, five months after its application, in Selvíria, Mato Grosso do Sul State, Brazil.

Treatments	SDM	SGY	DM
	kg ha^−1^		
Gypsum rates (G) (t ha^−1^)			
0	102,315	1919	3203
2.9	95,833	1720	2904
5.7	95,833	1779	2596
8.5	91,667	1700	2663
11.4	105,093	1768	2979
Inoculation (I)			
with	5721 a	1950 a	3138 a
without	3120 b	1630 b	2600 b
Pr > Fc (G)	0.8221 ^ns^	0.2354 ^ns^	0.9483 ^ns^
Pr > Fc (I)	0.0001 *	0.0001 *	0.0001 *
Pr > Fc (G × I)	0.3554 ^ns^	0.5412 ^ns^	0.3895 ^ns^
DMS	1123	120	515
CV (%)	20.7	18.8	16.1

Means followed by different letters in the column for different treatments differ according to Tukey’s test at 5% probability. *: significant at 5% probability^. ns^ not significant. Where CV is the coefficient of variation, and DMS is the least significant difference. SDM: Shoot dry matter production of sorghum; SGY: Sorghum grain yield and DM: Forage dry matter production.

**Table 4:** Maize grain yield (MGY) and Massai grass dry matter production (MDM) during the maize–Massai grass intercropping system from 12/2018 to 05/2019, as a function of the effects of *A. brasilense* inoculation and residual gypsum 13 months after application.

Treatments	MGY	MDM
	kg ha^−1^	
Gypsum rates (G) (t ha^−1^)		
0	3738	1984
2.9	5617	2321
5.7	4063	2207
8.5	4289	2190
11.4	3597	2498
Inoculation (I)		
with	3990	2251
without	4531	2229
Pr > Fc (G)	0.001 *	0.002 *
Pr > Fc (I)	0.050 *	0.762 ^ns^
Pr > Fc (G × I)	0.016 *	0.002 *
DMS	541.27	149.51
CV (%)	19.58	10.28

*: significant at 5% probability. ^ns^ not significant. Where CV is the coefficient of variation and DMS is the least significant difference. MGY: Maize grain yield and MDM: Massai grass dry matter production.

**Table 5:** Dry matter yield of black oat from June 2019 to September 2019 (DMBO 1) and from May 2020 to August 2020 (DMBO 2) as affected by inoculation with *A. brasilense* and residual gypsum 18 and 29 months after application, respectively, in Selvíria, Mato Grosso do Sul State, Brazil.

Treatments	DMBO 1	DMBO 2
	kg ha^−1^	
Gypsum rates (G) (t ha^−1^)		
0	2045	10,095
2.9	1898	10,449
5.7	2361	9436
8.5	1925	9727
11.4	1911	9496
Inoculation (I)		
With	2270 a	10,228
Without	1786 b	9453
Pr > Fc (G)	0.440 ^ns^	0.518 ^ns^
Pr > Fc (I)	0.001 *	0.075 ^ns^
Pr > Fc (G × I)	0.115 ^ns^	0.235 ^ns^
DMS	466	1025
CV (%)	16.19	13.48

Means followed by different letters in the column for different treatments differ according to Tukey’s test at 5% probability. *: significant at 5% probability. ^ns^ not significant. Where CV is the coefficient of variation and DMS is the least significant difference. DMBO 1: Dry matter yield of black oat from June 2019 to September 2019; DMBO 2: Dry matter yield of black oat from May 2020 to August 2020.

**Table 6:** Soil chemical attributes in the 0–0.20 and 0.20–0.40 m layers before applying agricultural gypsum, Selvíria, State of Mato Grosso do Sul, Brazil, 2017.

Soil Depth	P-Resin	S-SO_4_^−^	OM	pH	K	Ca	Mg	H+Al	Al	SB	CEC	V	m
m	mg dm^−3^		g dm^−3^		mmol_c_ dm^−3^							%	
0.00–0.20	37	7.0	27	4.6	2.5	29	19	42	2.0	50.5	92.5	55	4
0.20–0.40	17	4.0	20	4.6	0.8	18	12	34	2.0	30.8	64.8	48	6

**Note:** Phosphorus resin (Presin), organic matter (OM), sulfur (S-SO_4_^−^), hydrogen potential (pH), potassium (K), calcium (Ca), magnesium (Mg), potential acidity (H+Al), aluminum (Al), sum of bases (SB), cation exchange capacity (CEC), base saturation (V%), and aluminum saturation (m). pH: was determined in CaCl_2_ (calcium chloride).

## Data Availability

The original contributions presented in this study are included in the article/Supplementary Material. Further inquiries can be directed to the corresponding author.

## Supplementary Materials

The following supporting information can be downloaded at https://www.mdpi.com/article/10.3390/plants14203230/s1. Table S1: Results of soil analysis in the 0.00–0.20 m layer, 18 months after the application of agricultural gypsum and the use of plant growth-promoting bacteria in rotation crops under soil fertility parameters, in the municipality of Selvíria, State of Mato Grosso do Sul, Brazil; Table S2: Results of soil analysis in the 0.00–0.20 m layer, 40 months after the application of agricultural gypsum and the use of plant growth-promoting bacteria in rotation crops under soil fertility parameters, in the municipality of Selvíria, State of Mato Grosso do Sul, Brazil. Table S3: Results of soil analysis in the 0.20–0.40 m layer, 18 months after the application of agricultural gypsum and the use of plant growth-promoting bacteria in rotation crops under soil fertility parameters, in the municipality of Selvíria, State of Mato Grosso do Sul, Brazil. Table S4: Results of soil analysis in the 0.20–0.40 m layer, 40 months after the application of agricultural gypsum and the use of plant growth-promoting bacteria in rotation crops under soil fertility parameters, in the municipality of Selvíria, State of Mato Grosso do Sul, Brazil. Table S5: Results of soil analysis in the 0.40–0.60 m layer, 18 months after the application of agricultural gypsum and the use of plant growth-promoting bacteria in rotation crops under soil fertility parameters, in the municipality of Selvíria, State of Mato Grosso do Sul, Brazil. Table S6: Results of soil analysis in the 0.40–0.60 m layer, 40 months after the application of agricultural gypsum and the use of plant growth-promoting bacteria in rotation crops under soil fertility parameters, in the municipality of Selvíria, State of Mato Grosso do Sul, Brazil.

## Abbreviations

The following abbreviations are used in this manuscript:

V%	Base saturation
NTS	No-tillage systems
Ca	Calcium
Mg	Magnesium
K	Potassium
PGPB	Plant growth-promoting bacteria
BNF	Biological nitrogen fixation
ICLS	Integrated crop–livestock production system
S–SO4	Sulfur
H+Al	Potential acidity
SB	Sun of bases
CEC	Cation exchange capacity
OM	Organic matter
P	Phosphorus
GY	Soybean grain yield
SDM	Sorghum dry matter
SGY	Sorghum grain yield
DM	Forage dry matter
MGY	Maize grain yield
MDM	Massai grass dry matter production
DMBO	Dry matter yield of black oat
