# Supplementary material for: Effects on Soil Fertility and Crop Productivity Under Residual Agricultural Gypsum and Azospirillum brasilense in Cover Crops in a Consolidated No-Tillage System
Source: Plants (Basel). 2025 Oct 21;14(20):3230. doi: 10.3390/plants14203230 (PMC12567402; doi:10.3390/plants14203230)
Supplement: Supplementary file 1 [file plants-14-03230-s001.zip › plants-3895766-supplementary-done.pdf]

plants

MDPI

Supplementary material

Effects on Soil Fertility and Crop Productivity under Residual Agricultural Gypsum and *Azospirillum brasilense* in Cover Crops in a Consolidated No-Tillage System

Isadora Nicolielo de Souza [1], Maria Eduarda Pafetti Cristovam [1], Eduardo Leandro Moraes [1], Viviane Cristina Modesto [1], Naiane Antunes Alves Ribeiro [1], Vitória Almeida Moreira Girardi [1], Nelson Câmara de Souza Júnior [1], Aline Marchetti Silva Matos [1], Jussara Souza Salles [1], Wander Luís Barbosa Borges [2], Marcelo Andreotti [1]

[1] Department of Plant Health, Rural Engineering and Soils, College of Engineering, São Paulo State University—UNESP-FEIS, Ilha Solteira 15385-088, SP, Brazil; isadora.nicolielo@unesp.br; maria.pafetti@unesp.br; el.moraes@unesp.br; viviane.modesto@unesp.br; na.antunes@unesp.br; vitoria.almeida@unesp.br; aline.marchetti@unesp.br; jussara-souza.salles@unesp.br; marcelo.andreotti@unesp.br

[2] Advanced Research Center for Rubber Tree and Agroforestry Systems, Agronomic Institute (IAC), Votuporanga 15505-970, SP, Brazil; wander.borges@sp.gov.br

* Correspondence: souza.jr@unesp.br**Table S1.** Results of soil analysis in the 0.00–0.20 m layer, 18 months after the application of agricultural gypsum and the use of plant growth-promoting bacteria in rotation crops under soil fertility parameters, in the municipality of Selvíria, State of Mato Grosso do Sul, Brazil.

Academic Editor(s): Name

Received: date
Revised: date
Accepted: date
Published: date

Citation: de Souza, I.N.; Cristovam, M.E.P.; Moraes, E.L.; Modesto, V.C.; Ribeiro, N.A.A.; Girardi, V.A.M.; de Souza Júnior, N.C.; Matos, A.M.S.; Salles, J.S.; Borges, W.L.B.; et al. Effects on Soil Fertility and Crop Productivity under Residual Agricultural Gypsum and *Azospirillum brasilense* in Cover Crops in a Consolidated No-Tillage System. *Plants* **2025**, *14*, x. https://doi.org/10.3390/xxxxx

Copyright: © 2025 by the authors. Submitted for possible open access publication under the terms and conditions of the Creative Commons Attribution (CC BY) license (https://creativecommons.org/licenses/by/4.0/).

Table S1. Results of soil analysis in the 0.00–0.20 m layer, 18 months after the application of agricultural gypsum and the use of plant growth-promoting bacteria in rotation crops under soil fertility parameters, in the municipality of Selvíria, State of Mato Grosso do Sul, Brazil.

0.00-0.20 m												
TRET	P-resin	S-SO_4	OM	pH	K	Ca	Mg	H+Al	Al	SB	CEC	V
	------mg dm^{-3}------		g dm^{-3}	$CaCl_2$	------------------------------mmol$_c$ dm^{-3}------------------------------							%
without	33.0	20.5 a	22.0	4.7 b	1.2	16.9 b	8.7 b	40.8 a	5.2 a	25.8 b	67.6 b	39.8 b
with	42.0	10.1 b	22.0	5.0 a	1.5	25.3 a	14.2 a	34.1 b	2.3 b	41.9 a	76.0 a	53.2 a
0	36.0	13.9	21.0	4.7	1.4	18.8	9.9	43.1	5.9	29.0	71.1	38.9
2.9	35.0	13.7	21.0	4.9	1.3	20.5	11.6	37.4	4.0	33.3	66.7	43.4
5.7	39.0	16.7	23.0	5.0	1.1	24.0	12.1	36.5	3.1	37.3	74.8	49.0
8.5	36.0	18.0	21.0	4.6	1.5	19.6	10.8	37.6	3.2	30.6	65.0	43.4
11.4	40.0	14.3	25.0	5.1	1.4	22.0	12.5	32.6	1.5	31.9	81.5	58.9
Pr > Fc (I)	0.12^{ns}	0.02*	0.18^{ns}	0.01*	0.22^{ns}	0.001*	0.002*	0.01*	0.03*	0.001*	0.001*	0.02*
Pr > Fc (G)	0.35^{ns}	0.38^{ns}	0.45^{ns}	0.10^{ns}	0.52^{ns}	0.15^{ns}	0.13^{ns}	0.30^{ns}	0.14^{ns}	0.26^{ns}	0.20^{ns}	0.33^{ns}
Pr > Fc (I x G)	0.58^{ns}	0.49^{ns}	0.67^{ns}	0.22^{ns}	0.81^{ns}	0.31^{ns}	0.71^{ns}	0.52^{ns}	0.37^{ns}	0.42^{ns}	0.33^{ns}	0.68^{ns}
CV(%)	23.9	24.9	13.3	7	24.9	26.7	23.5	20.6	25.2	11.9	12.7	23.0

Note: P-resin: phosphorus; S-SO_4: sulfur; OM: organic matter; pH: hydrogen potential; K: potassium; Ca: calcium; Mg: magnesium; H+Al: potential acidity; Al: aluminum; SB: sum of bases; CEC: cation exchange capacity; V: base saturation; TRET: treatments; CV(%): coefficient of variation. Means followed by different letters in the column for different treatments differ according to Tukey's test at 5% probability. *: significant at 5% probability; ns: not significant.

Table S2. Results of soil analysis in the 0.00–0.20 m layer, 40 months after the application of agricultural gypsum and the use of plant growth-promoting bacteria in rotation crops under soil fertility parameters, in the municipality of Selvíria, State of Mato Grosso do Sul, Brazil.

0.00-0.20 m												
TRET	P-resin	$S\text{-}SO_4$	OM	pH	K	Ca	Mg	H+Al	Al	SB	CEC	V
	------mg dm^{-3}----		g dm^{-3}	$CaCl_2$	------------mmolc dm^{-3}------------							%
**Inoculation (I)**												
without	40.4	10.3 a	30.6 a	5.1	3.7 a	34.9	18.7	38.6	0.9	55.3	93.8	56.6
with	33.4	7.2 b	25.9 b	5.3	2.4 b	40.3	22.2	35	1.6	64.8	99.8	61.8
**Gypsum rates (G)**												
0	45.5	8.8	27.3	5.3	2.9	44.3	24.3	36.4	2.0	71.4	107.8	61.4
2.9	31.3	7.9	31.5	5.4	3.3	35.9	22.6	34.3	0.6	61.8	96.1	63.4
5.7	40.9	8.9	28.8	5.1	2.3	37.1	19.4	37.3	1.1	58.8	96.0	58.0
8.5	38.4	9.8	28.1	4.9	2.8	28.0	15.4	43.1	1.8	46.2	89.3	51.0
11.4	28.3	9.4	28.6	5.3	3.9	37.6	20.36	32.9	0.8	62.1	95.0	62.1
**Values of Pr > Fc**												
Pr > Fc (I)	0.33^{ns}	0.006*	0.001*	0.25^{ns}	0.004*	0.23^{ns}	0.32^{ns}	0.40^{ns}	0.25^{ns}	0.31^{ns}	0.35^{ns}	0.36^{ns}
Pr > Fc (G)	0.54^{ns}	0.26^{ns}	0.16^{ns}	0.37^{ns}	0.09^{ns}	0.57^{ns}	0.55^{ns}	0.59^{ns}	0.65^{ns}	0.56^{ns}	0.48^{ns}	0.65^{ns}
Pr > Fc (I x G)	0.15^{ns}	0.71^{ns}	0.08^{ns}	0.36^{ns}	0.39^{ns}	0.27^{ns}	0.20^{ns}	0.36^{ns}	0.54^{ns}	0.26^{ns}	0.22^{ns}	0.31^{ns}
CV(%)	22.3	25.3	15.8	10.5	23.4	24.1	24.9	25.4	32.1	24.7	20.8	24.6

Note: P-resin: phosphorus; $S\text{-}SO_4$: sulfur; OM: organic matter; pH: hydrogen potential; K: potassium; Ca: calcium; Mg: magnesium; H+Al: potential acidity; Al: aluminum; SB: sum of bases; CEC: cation exchange capacity; V: base saturation; TRET: treatments; CV(%): coefficient of variation. Means followed by different letters in the column for different treatments differ according to Tukey's test at 5% probability. *: significant at 5% probability; ns: not significant.

Table S3. Results of soil analysis in the 0.20–0.40 m layer, 18 months after the application of agricultural gypsum and the use of plant growth-promoting bacteria in rotation crops under soil fertility parameters, in the municipality of Selvíria, State of Mato Grosso do Sul, Brazil.

0.20-0.40												
TRET	P-resin	$S\text{-}SO_4$	OM	pH	K	Ca	Mg	H+Al	Al	SB	CEC	V
	------mg dm^{-3}------		g dm^{-3}	$CaCl_2$	------------------------------------mmol$_c$ dm^{-3} ----------------------------------							%
without	12.0	38.5 a	16.4 a	4.9	1.0	14.3 b	7.5 b	30.0	3.4 a	22.7 b	52.7 b	42.9 b
with	13.0	30.5 b	17.5 b	5.1	1.1	19.4 a	10.9 a	27.0	1.5 b	31.3 a	58.3 a	53.3 a
0	11.0	33.3	16.6	4.7	1.0	15.6	8.7	31.0	3.5	21.4	52.4	41.0
2.9	12.0	34.6	16.0	5.1	1.0	15.6	8.9	25.3	1.9	25.5	50.7	50.1
5.7	13.0	34.0	17.7	5.0	1.1	19.1	9.7	30.0	2.7	30.5	60.5	50.1
8.5	11.0	35.0	16.9	5.0	1.0	17.3	9.4	28.0	2.1	26.6	54.6	47.6
11.4	15.0	35.5	17.5	5.0	1.0	20.5	9.5	28.3	2.0	31.0	59.3	51.6
Pr > Fc (I)	0.22^{ns}	0.02*	0.001*	0.08^{ns}	0.35^{ns}	0.001*	0.03*	0.38^{ns}	0.001*	0.01*	0.02*	0.001*
Pr > Fc (G)	0.38^{ns}	0.44^{ns}	0.33^{ns}	0.17^{ns}	0.38^{ns}	0.06^{ns}	0.09^{ns}	0.46^{ns}	0.36^{ns}	0.07^{ns}	0.41^{ns}	0.18^{ns}
Pr > Fc (I x G)	0.55^{ns}	0.63^{ns}	0.60^{ns}	0.26^{ns}	0.42^{ns}	0.12^{ns}	0.31^{ns}	0.81^{ns}	0.53^{ns}	0.08^{ns}	0.43^{ns}	0.39^{ns}
CV(%)	24.7	24.1	9.9	6.6	15.4	24.5	23.7	22.7	26.6	24.9	13.1	20.1

Note: P-resin: phosphorus; $S\text{-}SO_4$: sulfur; OM: organic matter; pH: hydrogen potential; K: potassium; Ca: calcium; Mg: magnesium; H+Al: potential acidity; Al: aluminum; SB: sum of bases; CEC: cation exchange capacity; V: base saturation; TRET: treatments; CV(%): coefficient of variation. Means followed by different letters in the column for different treatments differ according to Tukey's test at 5% probability. *: significant at 5% probability; ns: not significant.

Table S4. Results of soil analysis in the 0.20–0.40 m layer, 40 months after the application of agricultural gypsum and the use of plant growth-promoting bacteria in rotation crops under soil fertility parameters, in the municipality of Selvíria, State of Mato Grosso do Sul, Brazil.

	P- resin	$S\text{-}SO_4$	OM	pH	K	Ca	Mg	H+Al	Al	SB	CEC	V
	--------mg dm^{-3}-------		g dm^{-3}	$CaCl_2$	---mmolc dm^{-3}-----------------------------------							%
**Inoculation (I)**												
without	10.9	40.8 a	16.5	4.8 b	1.3	14.8 b	7.0 b	36.9 a	1.7 a	23.1 b	60.0 b	38.7 b
with	9.4	28.2 b	15.9	5.2 a	1.1	21.4 a	10.5 a	31.4 b	0.6 b	33.0 a	64.3 a	50.7 a
**Gypsum rates (G)**												
0	9.1	28.5	15.6	5.0	1.4	14.8	8.1	33.9	1.4	26.7	58.2	41.9
2.9	8.8	24.1	16.3	5.1	1.1	18.5	9.9	33.3	1.0	27.2	62.8	46.0
5.7	12.1	24.9	16.5	4.9	1.2	17.9	9.0	37.0	1.1	27.7	65.0	42.8
8.5	9.4	33.4	16.5	5.2	1.2	16.6	8.9	33.4	0.6	28.8	63.1	46.9
11.4	11.4	36.5	16.1	5.1	1.2	19.9	7.6	33.1	1.1	29.8	61.9	45.9
**Values of Pr > Fc**												
Pr > Fc (I)	0.21^{ns}	0.004*	0.23ns	0.01*	0.07^{ns}	0.001*	0.0001*	0.0009*	0.001*	0.0001*	0.04*	0.001*
Pr > Fc (G)	0.35^{ns}	0.56^{ns}	0.79ns	0.30^{ns}	0.50^{ns}	0.12^{ns}	0.36^{ns}	0.43^{ns}	0.90^{ns}	0.40^{ns}	0.32^{ns}	0.53^{ns}
Pr > Fc (I x G)	0.38^{ns}	0.70^{ns}	0.09ns	0.47^{ns}	0.15^{ns}	0.50^{ns}	0.67^{ns}	0.38^{ns}	0.97^{ns}	0.53^{ns}	0.19^{ns}	0.66^{ns}
CV(%)	22.9	25.3	9.72	5.41	24.56	22.84	25.59	13.71	24.8	21.9	10.2	15.71

Note: P-resin: phosphorus; $S\text{-}SO_4$: sulfur; OM: organic matter; pH: hydrogen potential; K: potassium; Ca: calcium; Mg: magnesium; H+Al: potential acidity; Al: aluminum; SB: sum of bases; CEC: cation exchange capacity; V: base saturation; TRET: treatments; CV(%): coefficient of variation. Means followed by different letters in the column for different treatments differ according to Tukey's test at 5% probability. *: significant at 5% probability; ns: not significant.

Table S5. Results of soil analysis in the 0.40–0.60 m layer, 18 months after the application of agricultural gypsum and the use of plant growth-promoting bacteria in rotation crops under soil fertility parameters, in the municipality of Selvíria, State of Mato Grosso do Sul, Brazil.

	0.40-0.60 m											
TRET	P-resin	$S\text{-}SO_4$	OM	pH	K	Ca	Mg	H+Al	Al	SB	CEC	V
	------mg dm^{-3}------		g dm^{-3}	$CaCl_2$	--------------------------------$mmol_c$ dm^{-3}-------------------------------							%
**Inoculação (I)**												
without	4.0 b	72.3 a	13.8 b	5.1	0.7 b	11.1 b	6.5 b	22.5	1.2	18.7 b	41.2 b	45.1 b
with	7.5 a	50.2 b	14.9 a	5.2	0.9 a	15.7 a	9.5 a	21.8	0.7	26.3 a	48.1 a	53.6 a
**Doses de gesso (t ha^{-1})**												
0	3.5	68.3	13.6 [1]	5.1	0.8	10.1	6.7	22.7	1.5	17.9	40.6	43.6
2.9	5.3	50.5	14.3	5.3	0.8	13.3	8.1	20.7	0.5	22.4	43.1	50.9
5.7	8.3	55.5	15.4	5.1	0.9	15.5	8.6	23.1	1.4	25.1	48.3	51.5
8.5	5.9	62.3	14.6	5.1	0.8	13.1	8.7	22.5	1.0	22.9	45.4	49.9
11.4	5.7	69.6	13.9	5.1	0.8	15.1	8.0	21.7	0.5	24.1	45.9	50.9
**Values of Pr > Fc**												
Pr > Fc (I)	0.03*	0.01*	0.01*	0.31^{ns}	0.001*	0.04*	0.001*	0.11^{ns}	0.23^{ns}	0.0001*	0.001*	0.001*
Pr > Fc (G)	0.06^{ns}	0.23^{ns}	0.001*	0.44^{ns}	0.33^{ns}	0.12^{ns}	0.20^{ns}	0.21^{ns}	0.52^{ns}	0.30^{ns}	0.38^{ns}	0.40^{ns}
Pr > Fc (I x G)	0.22^{ns}	0.44^{ns}	0.28^{ns}	0.49^{ns}	0.50^{ns}	0.33^{ns}	0.41^{ns}	0.47^{ns}	0.70^{ns}	0.25^{ns}	0.52^{ns}	0.88^{ns}
CV(%)	27.1	34.6	10.8	6.1	16.8	23.9	25.4	14.9	27.1	26.9	15.9	14.1

Note: P-resin: phosphorus; $S\text{-}SO_4$: sulfur; OM: organic matter; pH: hydrogen potential; K: potassium; Ca: calcium; Mg: magnesium; H+Al: potential acidity; Al: aluminum; SB: sum of bases; CEC: cation exchange capacity; V: base saturation; TRET: treatments; CV(%): coefficient of variation. Means followed by different letters in the column for different treatments differ according to Tukey's test at 5% probability. *: significant at 5% probability; ns: not significant; [1] $y = 13.7 + 0.939x - 0.153x^2$ ($R^2 = 0.80$).

Table S6. Results of soil analysis in the 0.40–0.60 m layer, 40 months after the application of agricultural gypsum and the use of plant growth-promoting bacteria in rotation crops under soil fertility parameters, in the municipality of Selvíria, State of Mato Grosso do Sul, Brazil.

0.40-0.60 m												
TRET	P-resin	$S\text{-}SO_4$	OM	pH	K	Ca	Mg	H+Al	Al	SB	CEC	V
	mg dm^{-3}		g dm^{-3}	$CaCl_2$	mmolc dm^{-3}						mmolc dm^{-3}	%
**Inoculation (I)**												
without	7.7	52.8 a	15.4	5.0 b	1.1	13.9 b	6.9 b	32.2 a	1.5 a	21.9 b	54.1	40.6 b
with	5.7	40.2 b	14.3	5.3 a	1.0	18.5 a	9.3 a	27.1 b	0.0 b	28.7 a	55.8	51.0 a
**Gypsum rates (G)**												
0	6.4	41.1	14.6	5.1	1.2	13.5[1]	7.9	29.9	1.0	22.6[2]	52.4	43.1[3]
2.9	5.5	40.9	14.4	5.2	1.0	15.3	8.3	28	0.5	24.4	52.4	46.0
5.7	8.5	37.1	15.4	5.0	1.0	16.4	8.5	31.5	1.8	25.9	57.4	45.0
8.5	6.1	41.4	15.1	5.2	1.1	17.8	8.3	29.8	0.9	27.1	56.8	47.3
11.4	6.8	44.5	16.6	5.2	1.0	18.1	7.4	29.1	0.5	26.5	55.6	47.5
**Values of Pr > Fc**												
Pr > Fc (I)	0.15ns	0.004*	0.21ns	0.001*	0.16ns	0.001*	0.0004*	0.0001*	0.001*	0.01*	0.35ns	0.001*
Pr > Fc (G)	0.46ns	0.95ns	0.70ns	0.29ns	0.55ns	0.03*	0.77ns	0.32ns	0.77ns	0.26ns	0.28ns	0.35ns
Pr > Fc (G x I)	0.91ns	0.92ns	0.68ns	0.49ns	0.22ns	0.32	0.87ns	0.74ns	0.77ns	0.38ns	0.48ns	0.56ns
CV(%)	25.6	26.2	10.5	4.6	20.81	19	23.26	10.9	25.3	17.2	10.6	10.3

Note: P-resin: phosphorus; $S\text{-}SO_4$: sulfur; OM: organic matter; pH: hydrogen potential; K: potassium; Ca: calcium; Mg: magnesium; H+Al: potential acidity; Al: aluminum; SB: sum of bases; CEC: cation exchange capacity; V: base saturation; TRET: treatments; CV(%): coefficient of variation. Means followed by different letters in the column for different treatments differ according to Tukey's test at 5% probability. *: significant at 5% probability; ns: not significant; [1]$y = 13.58 + 1.70x - 0{,}16x^2$ ($R^2 = 0.99$); [2]$y = 22.57 + 2.11x - 0{,}24x^2$ ($R^2 = 0.99$); [3]$y = 43.57 + 1.37x - 0.12x^2$ ($R^2 = 0.82$).
